# Plasmon-enhanced photoluminescence from MoS_2_ monolayer with topological insulator nanoparticle

**DOI:** 10.1515/nanoph-2021-0685

**Published:** 2022-01-21

**Authors:** Dikun Li, Hua Lu, Yangwu Li, Shouhao Shi, Zengji Yue, Jianlin Zhao

**Affiliations:** MOE Key Laboratory of Material Physics and Chemistry under Extraordinary Conditions, Key Laboratory of Light-Field Manipulation and Information Acquisition, Ministry of Industry and Information Technology, and Shaanxi Key Laboratory of Optical Information Technology, School of Physical Science and Technology, Northwestern Polytechnical University, Xi’an 710129, China; Center for Artificial-Intelligence Nanophotonics, School of Optical-Electrical and Computer Engineering, University of Shanghai for Science and Technology, Shanghai 200093, China

**Keywords:** light–matter interaction, localized surface plasmons, photoluminescence, topological insulators

## Abstract

Topological insulators (TI), as a kind of fantastic nanomaterial with excellent electrical and optical properties, have attracted particular attention due to the promising applications in optoelectronic devices. Herein, we experimentally demonstrated the interaction between light and molybdenum disulfide (MoS_2_) monolayer with an antimony telluride (Sb_2_Te_3_) TI nanoparticle. It was found that photoluminescence (PL) emission and Raman scattering signal can be boosted by 5 and 8 folds in MoS_2_ monolayer integrated with the TI nanoparticle, respectively. The measured and simulated dark-field scattering spectra illustrated that the enhancement of light–matter interaction could be derived from the generation of localized surface plasmons on the TI nanoparticle with distinctly boosted electric field. We also found that there exists a redshift of 5 nm for the enhanced PL peak, which could be attributed to the formation of trions in MoS_2_ induced by plasmon doping. This work would provide a new pathway for the applications of TI nanoparticles in the optoelectronics, especially light–matter interaction enhancement.

## 1 Introduction

Two-dimensional (2D) layered semiconductor nanomaterials with excellent optical, electrical, mechanical, and thermal properties have attracted broad attentions in electronics, optoelectronics, and photonics [1], [2], [3]. As a kind of typical 2D nanomaterials, transition-metal dichalcogenides (TMDCs) have found considerable applications in optoelectronic functionalities and devices, for instance photodetection [4, 5], modulation [6, 7], frequency conversion [8], and lasering [9]. The energy band gaps of TMDCs are abundant and of dependence on atomic layer numbers [10]. For example, molybdenum disulfide (MoS_2_) bulk has an indirect bandgap of about 1.29 eV, but MoS_2_ monolayer presents a direct bandgap of about 1.9 eV [11]. The special bandgap characteristics are beneficial for the realization of the field effect transistors with an ultra-high on/off ratio [12]. Recently, MoS_2_ monolayer with attractive properties of light absorption, chemical stability, photoemission, and excitonic binding injects new vitality into solar cells [13], transistors [14], photocatalysis [15], and light emission [16, 17], and so on. However, the challenge facing us is that the atomic-layer structure of MoS_2_ with weak light–matter interaction prohibits the development of high-performance optoelectronic functionalities, for example photoluminescence (PL) emission [18]. Improving light interaction with MoS_2_ is crucial for highly efficient PL emission.

Different from traditional insulators/semiconductors and metals, topological insulators (TIs) are electronic materials with a bulk bandgap insulator and topologically protected conducting (metal-like) states on their edge or surface [19]. Due to the spin–orbit coupling effect in the bulk, the Dirac cores without the bandgap are protected by time-reversal symmetry. These unique properties contribute to the observation of exotic physical phenomena, such as quantum spin Hall effect [20], carrier backscattering avoidance [21], and Majorana fermions [22]. Recently, the topological surface states have been found in three-dimensional (3D) Sb_2_Te_3_, Bi_2_Te_3_, and Bi_2_Se_3_ materials [23]. These 3D materials exhibit unique electronic and optical features containing broad operating wavelength range, external tunability, and compatibility with optical elements [24], and play an important role in the realization of photonic Weyl points [25] and nanometric hologram [26]. Especially, the 3D TIs with conducting surface states enable the excitation of surface plasmons with lower loss and higher figure of merit in the ultraviolet and partly visible ranges compared with traditional metals [27, 28]. TIs with the generation of surface plasmons offer a new platform for the enhancement of light–matter interaction [29, 30].

Herein, we experimentally demonstrated the enhancement of interaction between light and MoS_2_ monolayer assisted by the Sb_2_Te_3_ topological insulator nanoparticle. The results show that the PL emission and Raman scattering can be enhanced by 5 and 8 folds in MoS_2_ monolayer with the TI nanoparticle, respectively. The dark field scattering spectrum denotes that the enhancement of light–MoS_2_ interaction results in the generation of localized surface plasmons with a strong electric field on the TI nanoparticle. The experimental measurement agrees well with the numerical simulations. A redshift is observed for the enhanced PL peak, which can be derived from the increased proportion of trions in MoS_2_ induced by plasmon doping. These results will pave a new avenue for exploring the enhancement of interaction between light and atomic-layer nanomaterials, especially for light emission.

## 2 Materials and methods

**Figure 1:**
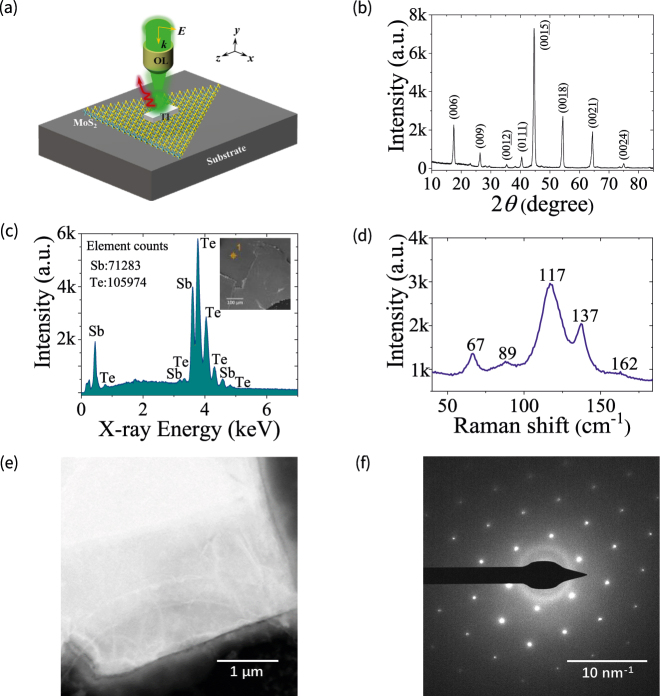
Structure and characterization of Sb_2_Te_3_ material. (a) 3D diagram of the MoS_2_ monolayer with a Sb_2_Te_3_ TI nanoparticle. (b) X-ray diffraction (XRD) patterns of Sb_2_Te_3_ crystal (aligned along the (001) plane) with a scanning angle range from 10° to 85° and a degree step of 0.02°. (c) X-ray spectrum of Sb_2_Te_3_ measured employing energy dispersive X-ray spectrometer (EDS) in scanning electron microscope (SEM). The inset shows the SEM top view of grown Sb_2_Te_3_ for EDS. (d) Raman scattering spectrum of Sb_2_Te_3_ excited with a 532 nm wavelength laser. (e) Transmission electron microscope (TEM) image in high-angle annular dark field (HAADF) mode of Sb_2_Te_3_. The scale bar is 1 μm. (f) Selected area electron diffraction (SAED) pattern of Sb_2_Te_3_. The scale bar is 10 nm^−1^.

The high-quality Sb_2_Te_3_ bulk material was grown from high purity Sb and Te powders with an atomic ratio of 2:3 by the melting-slow cooling method. The small Sb_2_Te_3_ bulk is placed on transparent tape and peeled repeatedly using the tapes until it is changed into particles. After this, we stick the tape onto the polydimethylsiloxane (PDMS) film, and peel the tape slowly enough to make the smaller particles stay on the PDMS. Then, the Sb_2_Te_3_ nanoparticle with suitable size is found and transferred to the MoS_2_ layer by using optical microscope and micromanipulation system. The MoS_2_ layer was grown on the silicon wafer in a quartz tube by using chemical vapor deposition (CVD) method. During the transfer process, the temperature of the transfer platform can be kept at about 60 °C. Figure 1(a) shows the 3D diagram of MoS_2_ layer with a TI nanoparticle. To characterize the crystalline and stoichiometry of TI material, we measured the X-ray diffraction (XRD, Shimadzu) pattern of Sb_2_Te_3_ bulk with a scanning angle range from 10° to 85° and a degree step of 0.02° by using Cu targets at 3 kW power, as can be seen in Figure 1(b). It is found that the XRD pattern is aligned along the (001) plane, which denotes that the Sb_2_Te_3_ material is single-crystalline. Figure 1(c) depicts the X-ray spectrum of the grown Sb_2_Te_3_ measured by energy dispersive X-ray spectrometer (EDS) in scanning electron microscope (SEM, FEI Verios G4). We can see that the material contains Sb and Te elements with a molar ratio of about 2:3. The inset of Figure 1(c) shows the SEM image of grown Sb_2_Te_3_. Moreover, we measured the Raman-shift spectrum of Sb_2_Te_3_ employing confocal Raman microscope (WITec alpha300R) with a 532 nm wavelength laser, as shown in Figure 1(d). The Raman peaks at 67, 89, 117, 137, and 162 cm^−1^ are consistent with those of Sb_2_Te_3_ single crystal [31]. The profiles of MoS_2_ and Sb_2_Te_3_ nanoparticle can be measured by using atomic force microscope (AFM, Bruker Dimension Icon) and SEM. The Raman shift spectra, dark-field scattering spectra, and PL emission of MoS_2_ layer with the TI nanoparticle were measured with confocal Raman microscope. In addition, transmission electron microscope (TEM, FEI Talos F200X) was used to further characterize the morphology and structure of Sb_2_Te_3_ material. TEM image in high-angle annular dark field (HAADF) mode and SAED pattern of Sb_2_Te_3_ are shown in Figure 1(e) and (f), respectively. These results verify the good single-crystal characteristic of Sb_2_Te_3_. The finite-difference time-domain (FDTD) method is used to numerically simulate the optical spectra and field distribution of the Sb_2_Te_3_ TI nanoparticle [32].

## 3 Results and discussion

**Figure 2:**
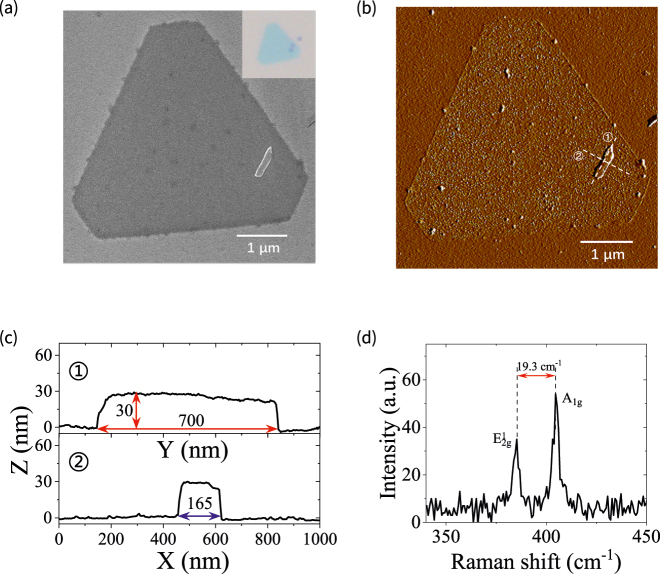
Characterization of MoS_2_ layer with the TI nanoparticle. (a) SEM top view of MoS_2_ layer with a Sb_2_Te_3_ TI nanoparticle and the corresponding optical microscopy image. (b) AFM-measured MoS_2_ layer with the Sb_2_Te_3_ TI nanoparticle. In (a) and (b), the scale bars are 1 μm. (c) Height profiles of Sb_2_Te_3_ nanoparticle along two white dotted lines in (b). (d) Raman shift spectrum of MoS_2_ layer excited with a 532 nm wavelength laser.

**Figure 3:**
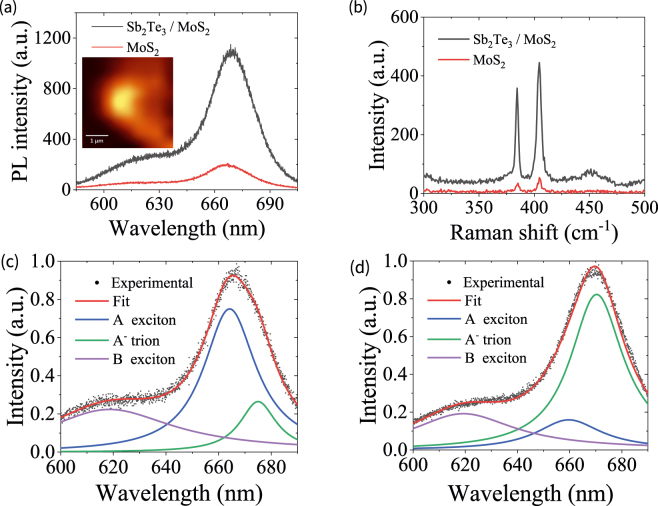
Enhanced PL emission and Raman shift spectra of MoS_2_ layer. (a) PL emission spectra of MoS_2_ monolayer with and without the Sb_2_Te_3_ TI nanoparticle. The inset shows the PL intensity mapping image around the Sb_2_Te_3_ TI nanoparticle. (b) Raman shift spectra of MoS_2_ monolayer with and without the Sb_2_Te_3_ TI nanoparticle. (c) Normalized PL spectra of A exciton, A^−^ trion, and B exciton in MoS_2_ monolayer on the substrate. (d) Normalized PL spectra of A exciton, A^−^ trion, and B exciton in MoS_2_ monolayer with the Sb_2_Te_3_ TI nanoparticle. The PL spectra in (c) and (d) are obtained by fitting the experimental data using the multi-Lorentzian function.

Figure 2(a) shows the SEM image of the CVD-grown MoS_2_ layer with a Sb_2_Te_3_ TI nanoparticle. The inset of Figure 2(a) depicts the optical microscopy image of the MoS_2_ layer with a Sb_2_Te_3_ TI nanoparticle. We measured the profile of the MoS_2_ layer with the TI nanoparticle, as shown in Figure 2(b). The AFM-measured profiles in Figure 2(c) show that the Sb_2_Te_3_ nanoparticle is approximately a rectangular shape with the length of *a* = 700 nm, width of *b* = 165 nm, and height of *h* = 30 nm. To clarify the MoS_2_ layer number, we measured the Raman shift spectrum of MoS_2_ layer with a 532 nm laser irradiation. It is shown that there exist obvious Raman peaks at about 385.3 and 404.6 cm^−1^, which correspond to the vibration mode E^1^_2g_ in the excitation plane and the vibration mode A_1g_ outside the plane of MoS_2_ monolayer, respectively [33]. The frequency difference between the two peaks is 19.3 cm^−1^, which is similar to the reported result of monolayer MoS_2_ [34]. Subsequently, we demonstrated the PL emission and Raman scattering of MoS_2_ monolayer with the TI nanoparticle for exploring the light interaction with MoS_2_ by using confocal Raman microscope with the objective lens of 100× and 532 nm excitation laser. As depicted in Figure 3(a), there exist two PL emission peaks at 620 and 665 nm wavelengths for MoS_2_ on the substrate, which corresponds to the B and A direct excitonic transitions of MoS_2_ monolayer at the K point of Brillouin zone [35]. The excitonic behaviors of PL emission in MoS_2_ may be changed under the condition of low temperature [36]. The inset of Figure 3(a) shows the PL intensity image obtained by integrating PL spectral intensities around the 665 nm wavelength at the mapping area (3.4 μm × 3.4 μm) covering the TI nanoparticle. We can see an obvious maximum PL intensity at the position of TI nanoparticle, which is improved by 5 folds compared to the MoS_2_ monolayer without the TI nanoparticle. The value is remarkable compared with the PL enhancement of CdSe/ZnS QDs on the Bi_2_Te_3_ nanoplate [30]. It is worth noting that the area of PL enhancement is larger than that of TI nanoparticle, which results in the limited laser beam with the diameter of ∼500 nm. When the size of TI nanoparticle is larger than the diameter of laser beam, the PL emission can also be enhanced around the TI particle due to the plasmonic generation at the side of TI particle. Figure 3(b) shows the Raman spectra of MoS_2_ monolayer with and without the TI nanoparticle. The Raman intensity is strongest at the center of TI nanoparticle, and decreases at the positions away from the TI center. The Raman scattering signal can be considerably enhanced by 8 folds for the MoS_2_ monolayer with the TI nanoparticle. It is also found in Figure 3(a) that the PL peak presents a redshift of 5 nm for the A exciton emission. To elucidate the reason, we fit the PL emission spectra by means of the multi-Lorentzian function, where a kind of quasiparticle state (i.e., A^−^ trion) can be considered [37]. Figure 3(c) shows the fitting results of normalized PL spectra of A exciton, A^−^ trion, and B exciton for the MoS_2_ monolayer without the TI nanoparticle. We can see that the A excitons dominate in the PL emission for MoS_2_ monolayer on the silicon substrate. There exists the lower proportion for A^−^ trions in MoS_2_ on the substrate at 675 nm wavelength. However, the proportion of A^−^ trions dramatically increases for MoS_2_ monolayer with the TI nanoparticle, as shown in Figure 3(d). This behavior could be derived from the electron doping of MoS_2_ from the TI nanoparticle with the radiation of the light field [37]. The increased A^−^ trions at the longer wavelength result in the redshift of PL emission peak. When the Sb_2_Te_3_ is covered on MoS_2_, the binding energies of excitons will decrease with the increase of relative dielectric constant on MoS_2_ [38]. This results in the blueshift of A exciton and A^−^ trion of MoS_2_ PL emission, as shown in Figure 3(c) and (d).

**Figure 4:**
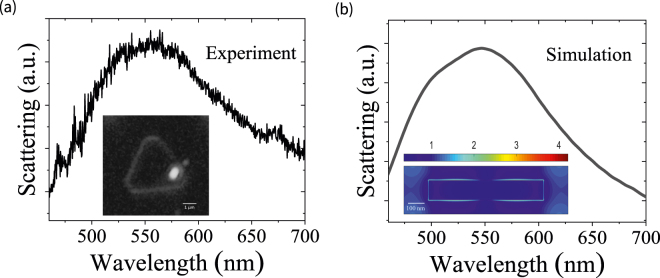
Dark-field scattering spectra of TI nanoparticle. (a) Experimentally measured dark-field scattering spectrum of the Sb_2_Te_3_ TI nanoparticle on the MoS_2_ monolayer. The inset shows the dark-field image of MoS_2_ monolayer with a Sb_2_Te_3_ TI nanoparticle. (b) Numerically simulated scattering spectrum of the Sb_2_Te_3_ TI nanoparticle. The inset shows the distribution of electric field |*E*| for the Sb_2_Te_3_ nanoparticle at the 532 nm wavelength. The scale bar is 100 nm. The experiment and numerical results are obtained by the confocal Raman microscope and FDTD simulation, respectively.

To clarify the mechanism of the above results, we measured the dark-field scattering spectrum of the Sb_2_Te_3_ TI nanoparticle on MoS_2_ monolayer using confocal Raman microscope, as shown in Figure 4(a). It is found that there exists a scattering spectral peak at the wavelength of 550 nm with a peak width of about 120 nm. The inset of Figure 4(a) depicts the dark-field image of the MoS_2_ monolayer with the Sb_2_Te_3_ TI nanoparticle. The scattering spectrum is achieved through dividing the scattering intensity from the sample by the incident light spectrum (measured by mirror reflector). To verify it, we numerically simulate the scattering spectrum of the Sb_2_Te_3_ TI nanoparticle using the FDTD method [32]. In FDTD simulations, we use an effective modeling method of dark-field scattering for the nanoparticles proposed by Jiang [39]. Two confocal Gaussian beams with a phase of π are used to construct an annular light source (dark-field light source), which is impinging on the Sb_2_Te_3_ TI nanoparticle. A power monitor with a finite area is set above the source. The perfectly matched layer absorbing boundary condition is set for the six planes in the computational area [39]. The relative permittivities of Sb_2_Te_3_ TI surface and bulk states are simultaneously considered in the simulations [40]. It is worth noting that both the surface and bulk states for Sb_2_Te_3_ present negative relative permittivities in the visible range of interest, which can contribute to the generation of surface plasmons. The thickness of TI surface layer is set as 2.6 nm [41]. The relative permittivity of silicon is set as the experiment data in Ref. [41]. The simulated scattering spectrum can be calculated by dividing the power passing through the monitor from the Sb_2_Te_3_ nanoparticle by that from the mirror reflector. As shown in Figure 4(b), the calculated scattering spectrum is in excellent agreement with the experimental result. The inset of Figure 4(b) depicts the simulated electric field distribution of |*E*| at 532 nm wavelength, which reveals the excitation of localized surface plasmons with a distinct field enhancement on the TI nanoparticle. The localized surface plasmons of TI nanoparticle can benefit for improving light–MoS_2_ interaction [30]. Therefore, it is understandable that the distinct enhancement of PL emission and Raman scattering can be observed in MoS_2_ monolayer with the TI nanoparticle [42]. The localized surface plasmons with the free-electron oscillation contribute to the doping of MoS_2_ monolayer with the TI nanoparticle, resulting in the increased A^−^ trions and PL peak intensity. Thus, the charge transfer will exist between the MoS_2_ monolayer with Sb_2_Te_3_ TI nanoparticle. The strain of MoS_2_ on the nanocone substrate will give rise to the redshift of Raman and PL emission peaks [43]. In our structure, the MoS_2_ grown on a flat substrate is covered on TI nanoparticle; the redshift of the PL peak induced by the strain can be neglected.

## 4 Conclusions

In this paper, we have demonstrated the interaction between light and MoS_2_ monolayer integrated with a Sb_2_Te_3_ TI nanoparticle. Through the fixed-point transfer method, the TI nanoparticle is transferred to the CVD-grown MoS_2_ monolayer. The experimental results show that the PL emission and Raman scattering intensity with a 532 nm excitation laser can be improved by 5 and 8 folds for MoS_2_ monolayer with the TI nanoparticle. The dark-field scattering spectrum illustrates that the TI nanoparticle can support the generation of localized surface plasmons with a distinct electric field enhancement. The experimental measurement is consistent with the FDTD numerical simulations. The improvement of PL emission and Raman scattering of MoS_2_ monolayer can be attributed to the formation of localized surface plasmons on the TI nanoparticle. The localized surface plasmons also contribute to the 5 nm redshift of PL emission peak with the increase of A^−^ trions proportion in MoS_2_ monolayer by plasmon doping. The crystal quality of MoS_2_ can be improved using the modified CVD growth method [44], [45], [46]. The improvement of MoS_2_ quality would enhance Raman and PL emission in the systems. This work provides a new pathway for the applications of TI materials in light–matter interaction and high-performance optoelectronic functionalities based on atomic-layer materials.
